# Sleep debt and depressive symptoms among workers: the mediating role of fatigue

**DOI:** 10.3389/fpubh.2026.1783461

**Published:** 2026-03-16

**Authors:** Chie Omichi, Yukiyoshi Sumi, Isa Okajima, Yuji Ozeki, Kojiro Ishii, Takaaki Mori, Uyanga Tsovoosed, Hiroshi Kadotani

**Affiliations:** 1 Organization for Research Initiatives and Development Doshisha University, and Research Center for Medical and Sports Science, Kyotanabe, Japan; 2 Department of Psychiatry, Shiga University of Medical Science, Otsu, Japan; 3 Department of Psychological Counseling, Faculty of Humanities, Tokyo Kasei University, Tokyo, Japan; 4 Faculty of Health and Sports Science, Doshisha University, Kyotanabe, Japan; 5 Graduate School of Health and Sports Science, Doshisha University, Kyotanabe, Japan; 6 Japan Society for the Promotion of Science, Tokyo, Japan; 7 Department of Communication Skills, Mongolian National University of Medical Sciences, Ulaanbaatar, Mongolia

**Keywords:** depression, fatigue, mediation analysis, occupational health, sleep debt

## Abstract

**Introduction:**

This study examined whether fatigue mediates the association between sleep debt and depressive symptoms among municipal employees in Japan.

**Methods:**

This cross-sectional study was conducted as part of the Night in Japan Home Sleep Monitoring Study (NinJaSleep Study) between November 2023 and March 2024 among 1,435 municipal employees in Koka City, Japan. Sleep debt was quantified using the Sleep Debt Index, calculated from self-reported ideal and actual sleep duration. Depressive symptoms were assessed using the Patient Health Questionnaire-9 (PHQ-9), and fatigue was assessed using the Chalder Fatigue Scale. Mediation analysis was performed using the PROCESS macro (Model 4), adjusting for age, sex, body mass index, and occupation. Indirect effects were estimated using bootstrapping with 5,000 resamples.

**Results:**

Sleep debt was significantly associated with higher fatigue scores (*B* = 2.37, *p* < 0.001), and fatigue was positively associated with depressive symptoms (*B* = 0.32, *p* < 0.001). The direct association between sleep debt and depressive symptoms was not statistically significant (*B* = 0.07, *p* = 0.36). Mediation analysis indicated that the association between sleep debt and depressive symptoms was significantly mediated by fatigue (indirect effect: *B* = 0.76, 95% CI: 0.61–0.91).

**Discussion:**

Sleep debt is associated with depressive symptoms primarily through increased fatigue. These findings highlight fatigue as a key pathway in linking insufficient sleep to mental health outcomes and underscore the importance of addressing fatigue in workplace mental health strategies.

## 1 Introduction

Sleep is essential for maintaining physical and mental health. However, in contemporary society, chronic sleep deprivation has become increasingly prevalent because of factors such as extended working hours, screen exposure, and irregular lifestyles. Japan, in particular, reports one of the shortest average sleep durations among the Organization for Economic Co-operation and Development (OECD) countries, at approximately 7 h and 22 min (1), which has raised concerns about the long-term health consequences of insufficient sleep.

Traditionally, studies have explored total sleep duration as a determinant of health, and numerous epidemiological findings have linked short sleep durations or insomnia to an increased risk of depressive symptoms (2, 3). Recently, the concept of *sleep debt*—it is defined as the discrepancy between ideal and actual sleep duration—has acquired significant academic attention (4). This measure may offer a more realistic and individualized indicator of sleep insufficiency, particularly in populations where self-imposed short sleep is common. Research indicates that accumulating sleep debt adversely impacts not only physical health, such as immune and metabolic functions, but also mental well-being (5–10).

A salient outcome of sleep debt is increased fatigue, which has been shown to compromise attention, emotional regulation, and performance in daily activities and work environments and is correlated with psychological distress (11, 12). Fatigue can induce decreased concentration and impaired emotional regulation, thereby increasing vulnerability to depressive symptoms (13, 14).

Additionally, fatigue has been linked to depression. Sunwoo et al. (2022), utilizing data from a large-scale national survey of the general population, reported that fatigue was significantly associated with depressive symptoms even after adjusting for other relevant factors (15). These findings, together with previous evidence indicating that fatigue is associated with substantial functional impairment, independent of specific psychiatric diagnoses (16), suggest that fatigue may serve as an important indicator of psychological distress. Given its high prevalence among working adults and substantial impact on daily functioning (17, 18), fatigue warrants attention as a clinically meaningful outcome in population-based studies.

Taken together, the evidence indicates that fatigue is a key pathway linking sleep disturbance to mental health outcomes. However, despite the increasing interest in sleep debt, empirical research on how fatigue mediates the relationship between sleep debt and depressive symptoms remains scarce. Ascertaining the pathways through which sleep debt leads to depression is the key to addressing rising mental health concerns among working adults. Exploring fatigue as a mediator may provide insights for improving sleep-related interventions and mental health strategies.

This study aimed to examine the association between sleep debt and depressive symptoms in a general population sample, focusing on the mediating role of fatigue. Clarifying the mechanisms underlying this association may provide a foundation for preventive measures that promote mental wellbeing through sleep management.

## 2 Methods

### 2.1 Participants and procedures

This study was conducted as part of the Night in Japan Home Sleep Monitoring Study (NinJaSleep Study), a cross-sectional survey investigating the relationship between sleep and mental health among workers. The survey was conducted in both paper- and web-based formats. The overall study framework and participant characteristics based on the NinJaSleep Study have been partially reported in previous publications (19, 20).

**Figure 1:**
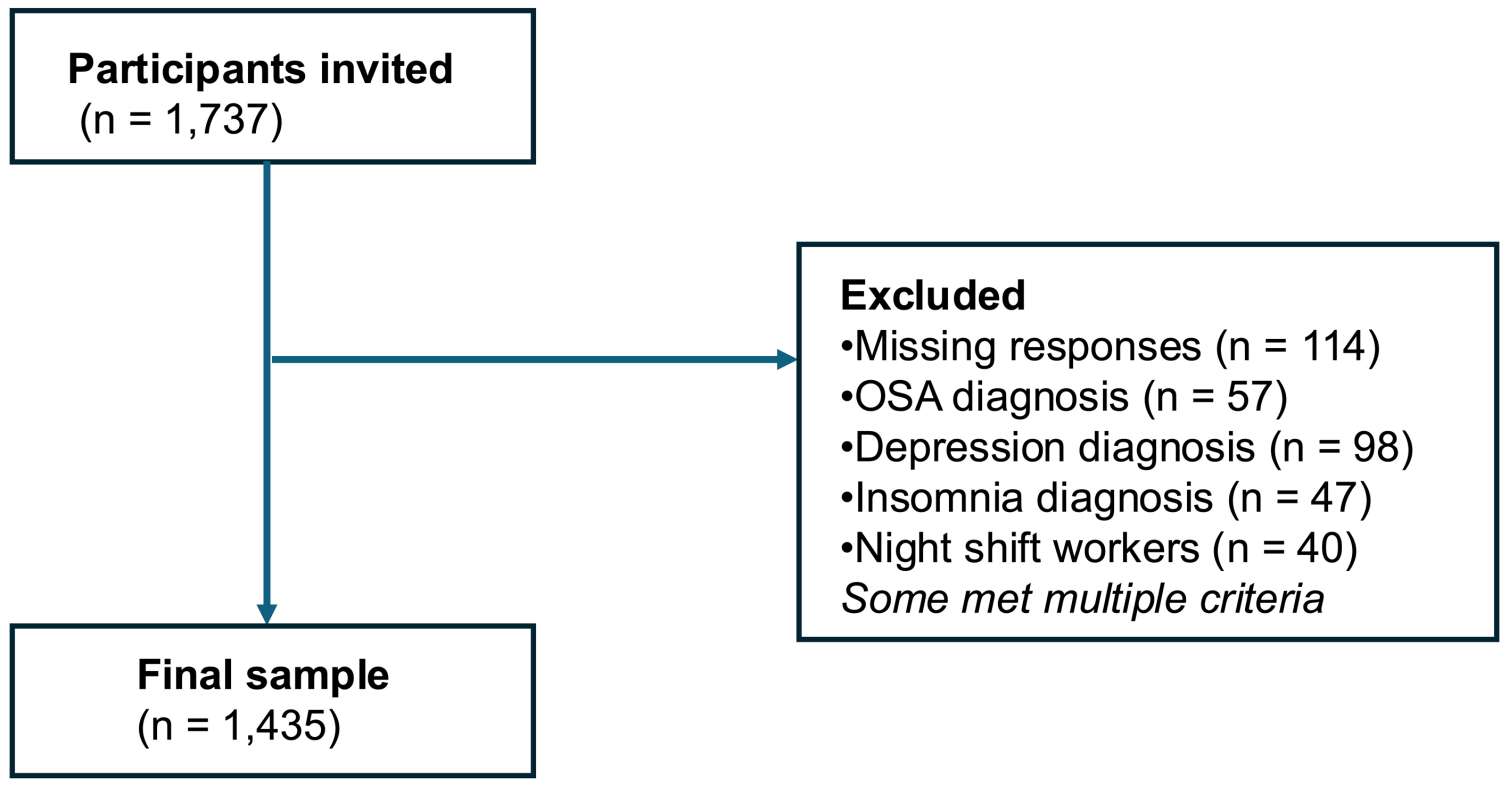
Participant flow.

In the present analysis, data were collected from municipal employees in Koka City, Shiga Prefecture, Japan, from November 2023 to March 2024. The flow of participant inclusion and exclusion criteria is shown in Figure 1. Of the 1,737 municipal employees invited to participate, participants were excluded for the following reasons: missing responses (*n* = 114), self-reported diagnosis of obstructive sleep apnea (OSA) (*n* = 57), diagnosis of depression (*n* = 98), diagnosis of insomnia (*n* = 47), and engagement in night shift work (*n* = 40). As some participants met more than one exclusion criterion, the final analytical sample consisted of 1,435 participants.

The study protocol was approved by the Ethics Committee of Shiga University of Medical Science (Approval No.: R2017-111), and informed consent was obtained from all participants. This study was registered at UMIN-CTR (UMIN000028675) and ClinicalTrials.gov (NCT03276585). The dataset analyzed in this study is available upon reasonable request from the corresponding author.

### 2.2 Measurements

Demographic variables including age, sex, body mass index (BMI), weight [kg]/height [m]^2^, occupation (administrative, technical, or other), smoking status (current smoker vs. nonsmoker), alcohol consumption frequency (0, 1–3, or ≥4 days/week), and comorbidities such as hypertension, dyslipidemia, diabetes, insomnia, and physician-diagnosed depression were assessed via self-report. Sleep-related measures included weekday and weekend sleep durations, as well as ideal sleep duration. Sleep debt was calculated using the Sleep Debt Index (SDI), which is defined as the average daily discrepancy between an individual’s ideal sleep duration and actual sleep duration. The actual sleep duration was calculated as the weighted average of weekday and weekend sleep durations as follows: actual sleep duration = (weekday sleep duration × 5 + weekend sleep duration × 2)/7. SDI = ideal sleep duration × actual sleep duration (if negative, the SDI was set to zero). This calculation follows the definition of the Sleep Debt Index (SDI) used in previous studies (4, 10). Additionally, sleep debt has been conceptualized as a deficit-based indicator of insufficient sleep (21); therefore, when the actual sleep duration exceeded the ideal sleep duration, sleep debt was interpreted as absent.

Psychosocial measures were assessed using standardized questionnaires. Depressive symptoms were evaluated using the Patient Health Questionnaire-9 (PHQ-9) (22), a self-administered questionnaire developed to assess the frequency of depressive symptoms experienced over the past 2 weeks. The scale consists of nine items, each rated on a 4-point Likert scale, with total scores ranging from 0 to 27. Higher scores indicate greater severity of depressive symptoms, and in the present study, a score of ≥10 was used as a cutoff value to indicate the presence of depressive symptoms (23). Fatigue was measured using the Chalder Fatigue Scale (CFS) (24, 25), a self-administered questionnaire developed to measure the severity of subjective physical and mental fatigue. The scale consists of 11 items, each rated on a 4-point Likert scale, assessing symptoms such as tiredness, lack of energy, and difficulties with concentration. The total fatigue score was calculated by summing all the items, with higher scores indicating greater fatigue severity. In this study, the CFS score was treated as a continuous variable.

### 2.3 Statistical analysis

All statistical analyses were performed using IBM SPSS Statistics version 30.0 (IBM Corp., Armonk, NY, USA) and the PROCESS macro version 5.0 for SPSS (Andrew F. Hayes, www.afhayes.com; Model 4). Statistical significance was set at *p* < 0.05. Continuous variables, including age, BMI, sleep duration, SDI, CFS, and PHQ-9, are summarized as mean ± standard deviation (SD), while categorical variables, such as sex and occupation, are presented as percentages. The distribution of SDI was examined, and normality was assessed using the Shapiro–Wilk test. Group comparisons were conducted using Student’s t-test for continuous variables and chi-square (*χ*^2^) tests for categorical variables to examine differences between age groups (<60 years vs. ≥60 years) and sex (men vs. women).

Mediation analysis was conducted using Hayes’ PROCESS macro (Model 4) to examine whether fatigue mediated the relationship between sleep debt and depressive symptoms. The indirect effects of sleep debt on depressive symptoms through fatigue were also tested. The significance of the indirect effect was assessed using 5,000 bootstrap samples to estimate at 95% confidence intervals (CI). The direct effect of sleep debt was also examined while controlling for covariates including age, sex, BMI, and occupation.

Additional logistic regression analyses were conducted to examine whether the mediation effects observed in the primary analysis were evident when depressive symptoms were treated as binary outcomes. The PHQ-9 score was dichotomized using a cutoff value of 10, with scores ≥10 classified as indicating the presence of depressive symptoms and scores <10 as indicating their absence. Furthermore, subgroup analyses were performed for individuals younger than 60 years and those aged 60 years and older, as this age cutoff is commonly used to distinguish working-age individuals from older adults in epidemiological research (26). This threshold was selected based on previous epidemiological studies and workforce classification standards in Japan, where the age of 60 often marks a transition to retirement or reduced work engagement. Additionally, sex-specific analyses were performed to examine the potential differences in the mediation pathways.

In addition to the primary cross-sectional analyses, supplementary longitudinal analyses were conducted to explore whether similar associations were observed over time. Detailed methods and results of the longitudinal analyses are provided in the Supplementary Materials (Supplementary Tables S1, S2).

## 3 Results

### 3.1 Descriptive statistics

Table 1 summarizes the demographic and sleep-related characteristics of participants. The mean age of the participants was 46.1 ± 13.1 years; 38.7% of the participants were men. The mean BMI was 22.3 ± 3.5 kg/m^2^. Regarding occupational distribution, 31.6% of the participants were in administrative roles, 39.9% in education or childcare, 2.0% in medical professions, 3.3% in manual labor, and 23.3% were contract-based workers.

**Table 1:** Descriptive characteristics of the study participants.

Variable	All (*n* = 1,435)	Men (*n* = 556)	Women (*n* = 879)	*p*-value	*y* < 60 (*n* = 1,176)	*y* ≥ 60 (*n* = 259)	*p*-value
	*	*	*		*	*	
Age (years)	46.1 ± 13.1	46.0 ± 14.5	46.2 ± 12.2	0.653	42.0 ± 10.6	64.9 ± 4.0	–
Men, *n* (%)	556 (38.7%)	–	–		426 (36.2%)	130 (50.2%)	**<0.001**
BMI (kg/m^2^)	22.3 ± 3.5	23.2 ± 3.3	21.8 ± 3.5	**<0.001**	22.2 ± 3.5	23.0 ± 3.4	**<0.001**
Job type, n (%)							
Administrative	453 (31.6%)	272 (48.9%)	181 (20.6%)	**<0.001**	410 (34.9%)	43 (16.6%)	**<0.001**
Education/Childcare	572 (39.9%)	193 (34.7%)	379 (43.1%)		509 (43.3%)	63 (24.3%)	
Medical	29 (2.0%)	8 (1.4%)	21 (2.4%)		24 (2.0%)	5 (1.9%)	
Manual labor	47 (3.3%)	21 (3.8%)	26 (3.0%)		16 (1.4%)	31 (12.0%)	
Contract-based	334 (23.3%)	62 (11.2%)	272 (30.9%)		217 (18.5%)	117 (45.2%)	
Current smoker	149 (10.4%)	127 (22.8%)	22 (2.5%)	**<0.001**	111 (9.4%)	38 (14.7%)	**0.008**
Drinking frequency (days/week)							
0 days (nondrinker)	821 (57.2%)	232 (41.7%)	589 (67.0%)	**<0.001**	687 (58.4%)	134 (51.7%)	**<0.001**
1–3 days	341 (23.8%)	145 (26.1%)	196 (22.3%)		300 (25.5%)	41 (15.8%)	
≥4 days	273 (19.0%)	179 (32.2%)	94 (10.7%)		189 (16.1%)	84 (32.4%)	
Comorbidity							
Hypertension	174 (12.1%)	91 (16.4%)	83 (9.4%)	**<0.001**	84 (7.1%)	90 (34.7%)	**<0.001**
Dyslipidemia	109 (7.6%)	48 (8.6%)	61 (6.9%)	0.141	56 (4.8%)	53 (20.5%)	**<0.001**
Diabetes	34 (2.4%)	20 (3.6%)	14 (1.6%)	**0.013**	11 (0.9%)	23 (8.9%)	**<0.001**
Sleep debt index (hours)	1.2 ± 1.0	1.1 ± 1.1	1.2 ± 1.0	0.19	1.3 ± 1.0	0.8 ± 0.8	**<0.001**
Weekday sleep duration (hours)	6.3 ± 1.0	6.3 ± 0.9	6.2 ± 1.0	**0.048**	6.3 ± 1.0	6.3 ± 1.0	0.216
Weekend sleep duration (hours)	7.3 ± 1.2	7.2 ± 1.2	7.3 ± 1.2	0.654	7.3 ± 1.2	7.0 ± 1.0	**<0.001**
Ideal sleep duration (hours)	7.7 ± 1.0	7.7 ± 1.0	7.7 ± 1.0	0.742	7.8 ± 1.0	7.3 ± 0.8	**<0.001**
PHQ-9 score	4.6 ± 4.4	4.1 ± 4.3	4.8 ± 4.5	**0.004**	5.0 ± 4.5	2.8 ± 3.3	**<0.001**
Depressive symptoms (PHQ-9 score≥10)	182 (12.7%)	62 (11.2%)	120 (13.7%)	0.095	171 (14.5%)	11 (4.2%)	**<0.001**
CFS score	15.3 ± 8.3	14.3 ± 8.6	16.0 ± 8.0	**<0.001**	16.0 ± 8.4	12.3 ± 7.1	**<0.001**

*Mean ± SD; SE, standard error; CI, confidence interval; SDI, Sleep Debt Index; PHQ-9, Patient Health Questionnaire-9; CFS, Chalder Fatigue Scale. *p*-values < 0.05 are shown in bold.

**Figure 2:**
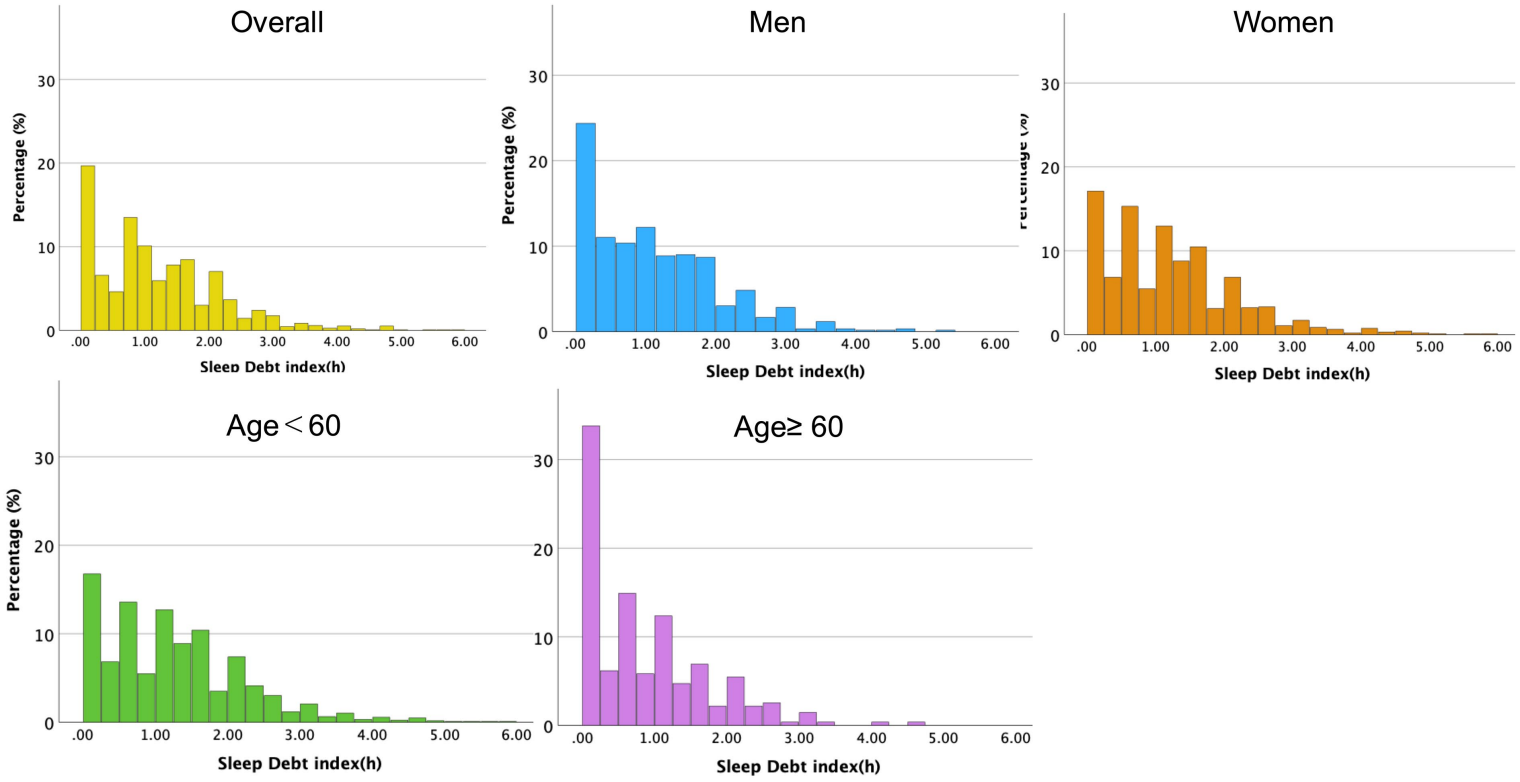
Histogram of sleep debt index (SDI) by gender and age group. Histograms display the distribution of SDI as a continuous variable, with the Y-axis indicating the percentage of participants within each SDI bin. Five plots are presented: overall, men only, women only, and gender-stratified plots for participants aged <60 and ≥60 years.

The mean SDI was 1.2 ± 1.0 h. Figure 2 depicts the distribution of the SDI according to sex and age. The mean sleep duration on weekdays was 6.3 ± 1.0 h, while on weekends it was 7.2 ± 1.2 h. The mean ideal sleep duration was 7.7 ± 1.0 h. The mean scores of PHQ-9 and CFS were 4.9 ± 4.8 and 15.3 ± 8.3, respectively.

Men showed higher smoking and drinking frequencies but lower PHQ-9 and CFS scores than women. Participants aged ≥60 years had lower SDI, PHQ-9, and CFS scores than those aged < 60 years.

### 3.2 Mediation analysis

**Figure 3:**
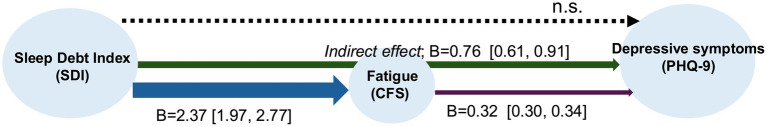
Mediation model illustrating the association between sleep debt and depressive symptoms through fatigue. Solid arrows represent statistically significant paths, whereas the dashed arrow indicates a non-significant direct association. The indirect effect via fatigue was statistically significant, as determined using 95% bootstrap confidence intervals.

Table 2 presents the results of the mediation analysis based on cross-sectional data from 2023 and Figure 3 illustrates the overall mediation model. Sleep debt was significantly associated with higher fatigue scores (*B* = 2.37, *p* < 0.001), and fatigue was positively associated with depressive symptoms (*B* = 0.32, *p* < 0.001). In contrast, the direct association between sleep debt and depressive symptoms was not significant (*B* = 0.07, *p* = 0.36).

**Table 2:** Path coefficients in the mediation model (cross-sectional data, 2023).

	Path	*B*	SE	*p*-value	95% CI (LL–UL)
All	SDI → CFS	2.37	0.20	**<0.001**	[1.97, 2.77]
	CFS → PHQ-9	0.32	0.01	**<0.001**	[0.30, 0.34]
	SDI → PHQ-9 (direct effect)	0.07	0.07	0.36	[−0.07, 0.21]
	SDI → CFS → PHQ-9 (indirect effect)	0.76	0.08	—	[0.61, 0.91]
Men	SDI → CFS	2.65	0.33	**<0.001**	[2.01, 3.29]
	CFS → PHQ-9	0.29	0.01	**<0.001**	[0.26, 0.31]
	SDI → PHQ-9 (direct effect)	0.07	0.11	0.54	[−0.15, 0.29]
	SDI → CFS → PHQ-9 (indirect effect)	0.76	0.12	—	[0.56, 1.01]
Women	SDI → CFS	2.16	0.26	**<0.001**	[1.65, 2.68]
	CFS → PHQ-9	0.34	0.01	**<0.001**	[0.32, 0.36]
	SDI → PHQ-9 (direct effect)	0.07	0.09	0.44	[−0.11, 0.26]
	SDI → CFS → PHQ-9 (indirect effect)	0.74	0.10	—	[0.55, 0.94]
*y* < 60	SDI → CFS	2.28	0.23	**<0.001**	[1.84, 2.72]
	CFS → PHQ-9	0.33	0.01	**<0.001**	[0.31, 0.35]
	SDI → PHQ-9 (direct effect)	0.06	0.08	0.49	[−0.10, 0.21]
	SDI → CFS → PHQ-9 (indirect effect)	0.75	0.08	—	[0.60, 0.92]
*y* ≥ 60	SDI → CFS	2.48	0.5	**<0.001**	[1.50, 3.46]
	CFS → PHQ-9	0.25	0.02	**<0.001**	[0.21, 0.29]
	SDI → PHQ-9 (direct effect)	0.11	0.16	0.51	[−0.21, 0.43]
	SDI → CFS → PHQ-9 (indirect effect)	0.61	0.16	—	[0.32, 0.95]

Covariates: age, sex, jobtype.

Model: PROCESS Model 4 (5,000 bootstrap samples, *n* = 1,435).

PHQ-9 scores were calculated excluding the fatigue item.

CFS fully mediated the association between subjective sleep debt and depressive symptoms.

*p*-values < 0.05 are shown in bold.

SE, standard error; CI, confidence interval; SDI, Sleep Debt Index; PHQ-9, Patient Health Questionnaire-9; CFS, Chalder Fatigue Scale.

The indirect effect of sleep debt on depressive symptoms through fatigue was statistically significant, as indicated by bootstrap confidence intervals (*B* = 0.76, 95% CI: 0.61–0.91), suggesting that fatigue fully mediated the association between sleep debt and depressive symptoms.

Similar mediation patterns were observed in sex- and age-stratified analyses, with significant indirect effects through fatigue in both men and women, as well as in participants younger than 60 years and those aged 60 years and above.

### 3.3 Additional analysis

Table 3 summarizes the results of the adjusted logistic regression analyses stratified by sex and age. SDI was significantly associated with depressive symptoms in the overall sample (OR = 1.64, 95% CI: 1.43–1.89, *p* < 0.001). Similar significant associations were observed in both men (OR = 1.61, 95% CI: 1.28–2.01) and women (OR = 1.67, 95% CI: 1.40–2.00). When stratified by age, SDI remained significantly associated with depressive symptoms in participants aged <60 years (OR = 1.60, 95% CI: 1.38–1.84) and ≥60 years (OR = 2.45, 95% CI: 1.23–4.88), with a stronger association observed in the older age group.

**Table 3:** Additional logistic regression analysis for depressive symptoms (OR and 95% CI).

Group	OR (SDI)	95% CI	*p*-value	OR (CFS)	95% CI (CFS)	*p*-value
All	1.64	(1.43, 1.89)	**<0.001**	1.31	1.26–1.36	**<0.001**
Men	1.61	(1.28, 2.01)	**<0.001**	1.29	1.22–1.37	**<0.001**
Women	1.67	(1.40, 2.00)	**<0.001**	1.33	1.27–1.40	**<0.001**
<60 years	1.60	(1.38, 1.84)	**<0.001**	1.30	1.25–1.35	**<0.001**
≥60 years	2.45	(1.23, 4.88)	**0.011**	1.54	1.24–1.91	**<0.001**

Covariates: age, sex, job type.

Model: adjusted logistic regression analysis.

*p*-values < 0.05 are shown in bold.

OR, odds ratio; CI, confidence interval. SDI, Sleep Debt Index; CFS, Chalder Fatigue Scale.

Fatigue, assessed using the CFS, was also independently associated with depressive symptoms across all strata. In the total sample, higher fatigue was associated with increased odds of depressive symptoms (OR = 1.31, 95% CI: 1.26–1.36, *p* < 0.001). This association was consistent in men (OR = 1.29, 95% CI: 1.22–1.37) and women (OR = 1.33, 95% CI: 1.27–1.40), as well as in participants aged <60 years (OR = 1.30, 95% CI: 1.25–1.35) and ≥60 years (OR = 1.54, 95% CI: 1.24–1.91).

## 4 Discussion

### 4.1 Summary of findings

This study examined the relationship between sleep debt and depressive symptoms, with emphasis on the mediating role of fatigue. The findings demonstrated that greater sleep debt was significantly associated with increased depressive symptoms, and that this association was largely mediated by fatigue. These results suggest that fatigue is a key mechanism by which sleep debt contributes to depressive symptoms.

### 4.2 Interpretation of findings

These results suggest that individuals with greater sleep debt experience heightened fatigue, which in turn exacerbates depressive symptoms. This aligns with previous research identifying sleep disturbance as a key contributor to mental health issues, particularly depression (27–29). In this context, the present findings underscore the role of fatigue as an important pathway through which sleep debt may be associated with depressive symptoms. Persistent fatigue may impair emotional regulation, cognitive functioning, and stress tolerance, thereby increasing the person’s vulnerability to depressive symptoms. (30, 31). Moreover, persistent fatigue has been linked to neurobiological mechanisms underlying depression, including dysregulation of the hypothalamic–pituitary–adrenal (HPA) axis and increased inflammatory responses (32). Thus, addressing fatigue may be a crucial step toward mitigating the mental health consequences of sleep debt.

### 4.3 Comparison with previous studies

Previous studies have demonstrated that sleep deprivation disrupts emotional regulation by weakening the functional control of the amygdala by prefrontal regions such as the ventral anterior cingulate cortex (vACC), resulting in heightened reactivity to negative emotional stimuli (33–37). These findings provide mechanistic insight into the effects of short-term sleep loss on emotional processing. Longitudinal studies have shown that persistent insomnia and short sleep duration, which reflect chronic sleep insufficiency, are associated with an increased risk of developing depression over time (38–40). Taken together, these studies indicate that insufficient sleep, both acute and chronic, plays an important role in emotional dysregulation and depression risk. Previous research has demonstrated that fatigue is strongly associated with depressive symptoms even after accounting for other sleep-related complaints, supporting the conceptualization of fatigue as a proximal factor linking sleep-related problems to mental health outcomes (15). Fatigue may reflect the cumulative burden associated with chronic sleep insufficiency (41, 42), and the present study suggests that accumulated sleep debt is associated with depressive symptoms, primarily through increased fatigue.

### 4.4 Individual differences: age and sex moderation effects

Our subgroup analyses revealed that the association between sleep debt and depressive symptoms was significant only among participants aged < 60 years and among women. Among younger and middle-aged workers, work-related stress and social responsibility may exacerbate the psychological impact of insufficient sleep. In contrast, older adults may be less affected by age-related declines in physiological sleep needs, which may lead to lower levels of perceived sleep insufficiency or fatigue despite short sleep duration (43).

The association between sleep debt and depressive symptoms was significant only in women. Furthermore, in women, the indirect effect of sleep debt on depressive symptoms through fatigue was significant, suggesting that fatigue may play a particularly important mediating role in the relationship between insufficient sleep and mental health. These findings are consistent with previous evidence indicating that women are more susceptible to depression, possibly due to hormonal fluctuations, complex social roles, and heightened sensitivity to sleep quality (44). Supporting this, a meta-analysis by Salk et al. (45) demonstrated that sex differences in depression emerged around 12 years of age and persisted throughout the lifespan, with women consistently exhibiting a higher prevalence than men.

### 4.5 Strengths and limitations

The two major strengths of this study include the sufficiently large sample size for effective analysis and the inclusion of municipal employees, which enabled a comprehensive examination of the association between sleep debt and mental health in the working population. In addition, the use of validated instruments to assess depressive symptoms and fatigue enhanced the reliability of our findings.

However, this study had several limitations should be acknowledged. First, although the primary analyses were cross-sectional, the causal inferences remained limited. While supplementary longitudinal analyses have provided supportive evidence, further prospective studies with longer follow-up periods are needed to more clearly establish the directionality and causality of the observed associations. Second, sleep debt was assessed using self-reported sleep duration, which may have been subject to recall bias and misclassification. Objective measures such as actigraphy or polysomnography can provide more accurate estimates of sleep patterns. Third, although adjustments were made for key confounders such as age, sex, BMI, and job type, this study did not assess certain lifestyle factors such as physical activity. Therefore, the influence of unmeasured factors on the results cannot be ruled out. Fourth, although sex was adjusted for in the multivariable analyses, the proportion of male participants was relatively low (38.7%), which may limit the generalizability of the findings, particularly to male-dominant occupational settings.

### 4.6 Implications for future research and practice

These findings suggest that the association between sleep debt and depressive symptoms operates primarily through fatigue, underscoring the importance of fatigue as a key intermediary linking insufficient sleep to mental health outcomes. Rather than viewing sleep debt as an isolated determinant, these results highlight the relevance of fatigue-related processes in understanding how insufficient sleep contributes to depressive symptoms in working adults. Practically, workplace approaches that address fatigue, such as ensuring adequate opportunities for recovery, introducing flexible work arrangements, and providing fatigue management education, may be beneficial in reducing the psychological burden associated with chronic sleep debt. Efforts to improve sleep hygiene may also play a supportive role by alleviating fatigue and promoting mental health.

Future studies, particularly those employing longitudinal or interventional designs, are warranted to examine whether reductions in sleep debt and fatigue translate into improvements in depressive symptoms. Clarifying the interrelationships among sleep debt, fatigue, and depressive symptoms may inform the development of more targeted and context-sensitive mental health strategies in both occupational and public health settings.

## 5 Conclusion

This study demonstrates that sleep debt is associated with depressive symptoms, primarily through increased fatigue. These findings highlight the central role of fatigue in linking insufficient sleep to mental health outcomes. Addressing chronic sleep deprivation and fatigue in occupational and public health contexts may help reduce the psychological burden on working populations and contribute to the promotion of mental well-being.

## Data Availability

The datasets presented in this article are not readily available because the datasets are subject to ethical and legal restrictions due to the inclusion of sensitive personal information and cannot be shared publicly. Requests to access the datasets should be directed to CO, comichi@mail.doshisha.ac.jp.
