# Supplementary material for: Sleep debt and depressive symptoms among workers: the mediating role of fatigue
Source: Front Public Health. 2026 Mar 16;14:1783461. doi: 10.3389/fpubh.2026.1783461 (PMC13055909; doi:10.3389/fpubh.2026.1783461)
Supplement: Supplementary file 1 [file Data_Sheet_1.pdf]

frontiers

Supplementary Material

1 Supplementary Results

Supplementary longitudinal analyses using adjusted logistic regression models examined the association between sleep debt at baseline (2022) and depressive symptoms (PHQ-9 $\geq$ 10) at follow-up in 2023 and 2024. Higher sleep debt at baseline was significantly associated with an increased odds of depressive symptoms at the 2023 follow-up (OR = 1.09, 95% CI: 1.02–1.17, $p = 0.012$). In contrast, the association was attenuated and no longer statistically significant at the 2024 follow-up (OR = 1.06, 95% CI: 0.97–1.15, $p = 0.208$). All the models were adjusted for age, sex, and job type. The Detailed results are presented in Supplementary Table S1.

A longitudinal mediation analysis was conducted to examine whether fatigue mediated the association between sleep debt at baseline (2022) and depressive symptoms at follow-up (2024). As shown in Supplementary Table S2, higher sleep debt in 2022 was significantly associated with higher fatigue scores in 2023 (B = 0.28, $p = 0.036$), and higher fatigue in 2023 was significantly associated with higher PHQ-9 scores in 2024 (B = 0.25, $p < 0.001$). The direct association between sleep debt in 2022 and depressive symptoms in 2024 was not statistically significant after accounting for fatigue (B = 0.07, $p = 0.18$). The indirect effect of sleep debt on depressive symptoms through fatigue was statistically significant based on bootstrap confidence intervals (95% CI: 0.001–0.17).

To reduce conceptual overlap between the mediator and outcome, the PHQ-9 score was calculated, excluding the item assessing fatigue (“feeling tired or having little energy”).

Table S1. Odds ratios for the association between baseline sleep debt (2022) and depressive symptoms at follow-up

**Variable**	**2023 Depressive symptoms (PHQ-9 ≥10)**			**2024 Depressive symptoms (PHQ-9 ≥10)**		
	OR	95% CI	p-value	OR	95% CI	p-value
2022 SDI	1.09	(1.02–1.17)	**0.012**	1.06	(0.97–1.15)	0.208

Covariates included age, sex, and job type. Results were obtained using logistic regression models.
Bold values indicate statistical significance ($p < 0.05$).
Abbreviations: SDI, sleep debt index; OR, odds ratio; CI, confidence interval.

Table S2. Longitudinal mediation analysis of the association between SDI (2022) and PHQ-9 (2024) via CFS (2023)

Path	Coefficient (B)	p-value	95% CI (LL–UL)
SDI (2022) → CFS (2023)	0.28	0.036	0.02–0.55
CFS (2023) → PHQ-9 (2024)	0.25	<0.001	0.22–0.28
SDI (2022) → PHQ-9 (2024) (direct)	0.07	0.18	−0.03–0.17
Indirect via CFS (2023)	0.07	—	0.001–0.17

Model: PROCESS Model 4 with 5,000 bootstrap samples.
Covariates: age, sex, and job type.
Note: PHQ-9 was calculated excluding the item *"feeling tired or having little energy"* to avoid conceptual overlap with the mediator (CFS).
Abbreviations: SDI, Sleep Debt Index; CFS, Chalder Fatigue Scale; PHQ-9, Patient Health Questionnaire; CI, confidence interval.
